# CRISPR/Cas9 Mediated Fluorescent Tagging of *Caenorhabditis elegans* SPE-38 Reveals a Complete Localization Pattern in Live Spermatozoa

**DOI:** 10.3390/biom13040623

**Published:** 2023-03-30

**Authors:** Yamei Zuo, Xue Mei, Andrew Singson

**Affiliations:** 1 Waksman Institute and Department of Genetics, Rutgers University, Piscataway, NJ 08854, USA; 2 Department of Biological Sciences, St. John’s University, Queens, New York, NY 11439, USA

**Keywords:** fertilization, *C. elegans*, sperm, *spe-9* class mutants, fertilization synapse, SPE-38, CRISPR/Cas9 genome editing

## Abstract

The *Caenorhabditis elegans spe-38* gene encodes a four-pass transmembrane molecule that is required in sperm for fertilization. In previous work, the localization of the SPE-38 protein was examined using polyclonal antibodies on spermatids and mature amoeboid spermatozoa. SPE-38 is localized to unfused membranous organelles (MOs) in nonmotile spermatids. Different fixation conditions revealed that SPE-38 either localized to fused MOs and the cell body plasma membrane or the pseudopod plasma membrane of mature sperm. To address this localization paradox in mature sperm, CRISPR/Cas9 genome editing was used to tag endogenous SPE-38 with fluorescent wrmScarlet-I. Homozygous male and hermaphrodite worms encoding SPE-38::wrmScarlet-I were fertile indicating the fluorescent tag does not interfere with SPE-38 function during sperm activation or fertilization. We found that SPE-38::wrmScarlet-I localized to MOs in spermatids consistent with previous antibody localization. In mature and motile spermatozoa we found SPE-38::wrmScarlet-I in fused MOs, the cell body plasma membrane, and the pseudopod plasma membrane. We conclude that the localization pattern observed with SPE-38::wrmScarlet-I represents the complete distribution of SPE-38 in mature spermatozoa and this localization pattern is consistent with a hypothesized role of SPE-38 directly in sperm-egg binding and/or fusion.

## 1. Introduction

Sperm and eggs form a fertilization synapse where proteins on the opposing gamete plasma membranes are hypothesized to form interacting protein complexes that mediate gamete binding and fusion [1]. Powerful forward and reverse genetic approaches have made great progress in identifying the molecular components of the fertilization synapse [2,3,4]. Determining the cellular location of these molecules will help determine their molecular functions and interactions.

**Figure 1:**
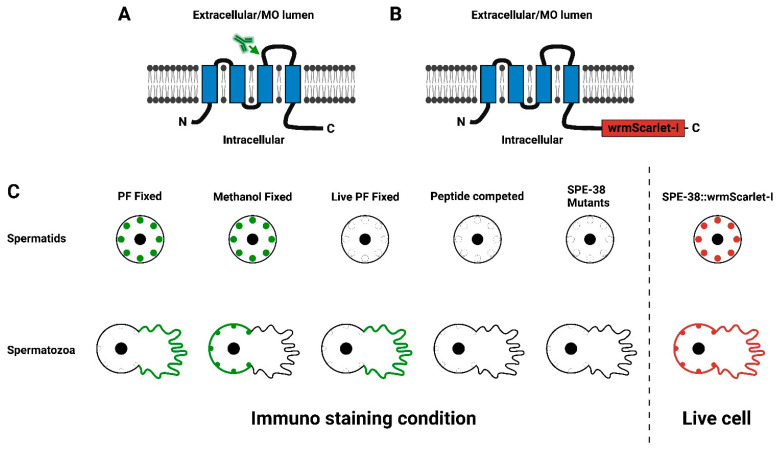
(**A**,**B**) A schematic representation of the *C. elegans* SPE-38. The four transmembrane domains are indicated by the blue boxes imbedded in the membrane. The N-Terminus and C-Terminus of SPE-38 are intracellular while the loops between the first and second transmembrane domain and between the third and fourth transmembrane domain are extracellular or in the MO lumen. The antibody symbol and arrow (**A**) indicate the location of the peptide sequence used to raise the polyclonal antisera used by Chatterjee et al., 2005. (**B**) A schematic representation of SPE-38::wrmScarlet-I. The red box (not to scale) indicates the location of wrmScarlet-I. The wrmScarlet-I sequence is codon optimized for expression in *C. elegans* along with key sequence changes (-I isoleucine substitution) that improve molecule stability. (**C**) The left panel is a schematic summary of previous immunolocalization experiments and controls for antisera specificity published in Chatterjee et al., 2005. Green indicates antibody staining in spermatids and mature sperm. In the right panel red indicates the observed distribution of SPE-38::wrmScarlet-I in spermatids and mature sperm.

Mutations in the *C. elegans spe-38* gene result in both male and hermaphrodite worms with a sperm-specific fertility defect [5]. Both hermaphrodite and male derived *spe-38* mutant sperm are indistinguishable from wild-type sperm in their morphology. These *spe-38* mutant sperm are also fully motile and can contact eggs at the site of fertilization in the worm reproductive tract. However, *spe-38* mutant sperm cannot enter the egg. Therefore, *spe-38* mutants display a “*spe-9* class” of sperm function defect [6]. The *spe-38* gene was positionally cloned and shown to encode a novel four-pass transmembrane molecule (Figure 1A) [5]. Although SPE-38 displays a four-pass membrane topology [5,7], it does not have significant amino acid sequence homology with tetraspanins such as CD9 [8]. It was found that SPE-38 can bind directly to numerous other *C. elegans* sperm membrane molecules that are required for functions such as sperm activation, fertilization, or egg activation [7,9]. Most notably, SPE-38 was found to bind to the sperm TRP-3/SPE-41 Ca^2+^-permeable channel. TRP-3/SPE-41 is thought to be required for fertilization as well as impact Ca^2+^ dynamics in the zygote [10,11,12].

To determine the subcellular localization of SPE-38, polyclonal rabbit antisera directed against a peptide corresponding to SPE-38 amino acids 101-114 (Figure 1A) was obtained by Chatterjee and coworkers [5]. This peptide sequence corresponds to a region in the large extracellular loop of SPE-38. The topology of SPE-38 was confirmed by yeast expression studies and live cell staining experiments [5,7,9]. These antisera when used for western blotting could detect a single band of approximately the predicted molecular weight of SPE-38 while no band was detected in *spe-38* null mutants. A diagrammatic summary of immunolocalization experiments and controls can be found in Figure 1C. The specificity of the antisera to SPE-38 was further confirmed by lack of staining on null mutant spermatids and spermatozoa. Finally, antisera preincubated with an excess of the 101-114 peptide lost all staining activity on spermatids and spermatozoa. In spermatids, regardless of fixation and permeabilization conditions, SPE-38 was detected in the unfused MOs of spermatids. As expected, the sera did not have access to SPE-38 in unfused MOs in live cell staining experiments. Further immunolocalization experiments demonstrated that both SPE-38 and TRP-3/SPE-41 co-localize to unfused membranous organelles (MOs) in round nonmotile spermatids [5,7]. During spermiogenesis, MOs fuse with the plasma membrane and provide a secretory pathway that alters the sperm surface in a way that is analogous to the acrosome reaction in sperm of other species [13,14]. TRP-3/SPE-41 moves from fused MOs and localizes to both the cell body and pseudopod of motile mature sperm [11] but remains sequestered in fused MOs in *spe-38* mutant sperm [7]. The functional requirement of SPE-38 for the movement of TRP-3/SPE-41 to the sperm surface and localization in the MO strongly indicates that SPE-38 has a role in moving other interacting sperm molecules to the sperm surface in this organelle.

In mature activated and motile sperm prepared with a methanol fixation protocol [5], SPE-38 was localized in MOs that were fused to the cell body plasma membrane and as well as the cell body plasma membrane (Figure 1C). No signal or very weak signal was detected on the pseudopod. In contrast, using a paraformaldehyde fixation or live cell staining protocols, SPE-38 was detected only on the pseudopod (Figure 1C). This fixation dependent localization paradox and its biological relevance with respect to SPE-38 function remained an unresolved issue.

Genome editing and powerful new microscopy tools made reexamining the localization of SPE-38 possible. In *C. elegans,* tagged fluorescent proteins have traditionally been introduced in by two methods. The oldest methods were by injecting DNA to form extrachromosomal arrays [15,16] or microparticle bombardment [17,18]. Poor expression levels of sperm transgenes almost always made visualization impossible [19,20]. Further, gene copy number from injection or bombardment were not well controlled and these typically highly repetitive arrays were subject to germline silencing and inconsistent inheritance [21]. A more recent method is MosSCI-based genetic integration systems [22]. While often effective for germline expressed tagged molecules, artifacts could arise due to non-physiological levels of gene expression and lack of endogenous regulatory control [23]. Recent advances in CRISPR/Cas9 genome editing tools have now allowed fluorescent tagging of endogenous genes under their native regulation [24]. This approach has allowed the successful mNeonGreen tagging of a highly expressed sperm gene [20] as a proof of concept. Advances in creating highly stable, species optimized codon usage, and bright GFP-like fluorescent tags as well as advances in microscopy [25,26,27,28,29,30] have helped transform our ability to visualize *C. elegans* sperm proteins.

A complete understanding of the function of SPE-38 depends on knowing its full subcellular location. Here we report the reexamination of SPE-38 distribution in live spermatids as well as mature and motile spermatozoa utilizing new technologies. We resolve a distribution paradox that arose due to differential fixation conditions used in previous immunolocalization studies. We confirm the localization of SPE-38 to unfused MOs in spermatids. This distribution of SPE-38::wrmScarlet-I in live cells is consistent with a function in helping move interacting molecules to the sperm surface from MOs. In motile live sperm, we detect SPE-38::wrmScarlet-I in fused MOs, the cell body plasma membrane, and the pseudopod plasma membrane. Plasma membrane localization places SPE-38 on the surface of mature sperm where it could directly interact with additional sperm and egg surface proteins during fertilization.

## 2. Materials and Methods

### 2.1. Worm Strains, Brood Sizing, Statistical Analysis, Sperm Preparations, and C. elegans Culture

*C. elegans* strains were bred and maintained using standard techniques [31]. All experiments were conducted with 20 °C culture conditions. Bristol N2 was the wild-type strain. All other strains were N2 derived. In numerous experiments the *him-5(e1490)* genetic background was used that increases the frequency of males. This genetic background does not cause adverse effects on sperm [13,19]. The fertility of hermaphrodite and male CRISPR/Cas9 genome edited strains and isolation and activation of sperm was as previously reported [5,32]. For hermaphrodite fertility analysis, L4 larval stage individual hermaphrodites were placed on single culture plates and transferred to fresh plates daily. Total progeny for 5 days of adulthood was quantified. For male fertility analysis, 3 L4 stage males were mated to single hermaphrodites for 24 h and outcrossed progeny counts were collected for the first 2 days. The *dpy-11(e224)* mutation in hermaphrodites was used as a recessive paternity marker for male fertility experiments. A two tailed unpaired Student’s *t*-test was used for statistical comparisons between wild-type control worms and genome edited worm brood sizes using GraphPad Prism 9.2.0 software. Male worms were dissected for the imaging of spermatids as previously described [33]. Males were isolated at L4 stage on culture plates and spermatids were dissected in sperm media (SM) plus dextrose for imaging after celibacy for 1 day. In vivo activated spermatozoa were dissected from *him-5(e1490)*; *spe-38::wrmScarlet-I* hermaphrodites that were mated to *him-5(e1490)*; *spe-38::wrmScarlet-I* males overnight. Identical crosses with *him-5(e1490)* were used as controls. Dissected hermaphrodites released in vivo activated spermatozoa derived from the hermaphrodite or males with the same genotype.

### 2.2. CRISPR/Cas9 Mediated Fluorescent Tagging of Endogenous SPE-38

SunyBiotech (15F/IFC, 1, Wang Long Er Rd, Tai Jiang District, Fuzhou, Fujian, China 350004) was consulted on design and contracted to construct our CRISPR/Cas9 genome edited animals. An in-frame C-terminal wrmScarlet-I tag was inserted in the native *spe-38* gene prior to the endogenous stop codon. The gRNA sequences used for specific and successful CRISPR/Cas9 insertion were “CCTGGAGTATGAGGCGGATTTGG” and “GGATGAGAAATCAGAGAAGAAGG”. Two synonymous nucleotide changes were generated in the donor sequence to prevent the donor from being recognized and cut by Cas9. The changes were made from “AATTCCTGGAGTACGAGGCGGATTTGGATGAGAAATCAGAGAAGAAGGATTAA” to “AATTCCTGGAGTACGAAGCGGATTTGGATGAGAAATCAGAGAAGAAAGAT-wrmScarlet-I sequence-TAA”. This fluorescent tag was chosen for its compatibility with polytopic transmembrane molecules and its specific fluorescent properties [27]. Genome edited animals were sequence verified and backcrossed to wild-type N2 worms several times to reduce the chances of extraneous genetic variation or off target insertions impacting our experiments. After backcrossing with N2 the endogenously tagged strain was sequence verified with *spe-38* specific primers (CCAAACTTCAGAATCTCAATGCG and TCGAGCTTATGAGACCTGTTC). The AD319 strain: *spe-38(syb6556[spe-38::wrmScarlet-I])*; *him-5(e1490)* will be deposited in the Caenorhabditis Genetics Center (CGC).

### 2.3. Microscopic Imaging of SPE-38::wrmScarlet-I Localization in Spermatids and Spermatozoa

Male worms were dissected and the spermatids or spermatozoa were imaged by bright field or 561 nm laser illumination using a Zeiss Elyra 7 inverted confocal microscope equipped with a 63× objective with water immersion. Images were processed with structured illumination microscopy (SIM) on Zeiss Elyra 7 Zen Black software.

## 3. Results

### 3.1. Genome Edited Animals Are Fertile

**Figure 2:**
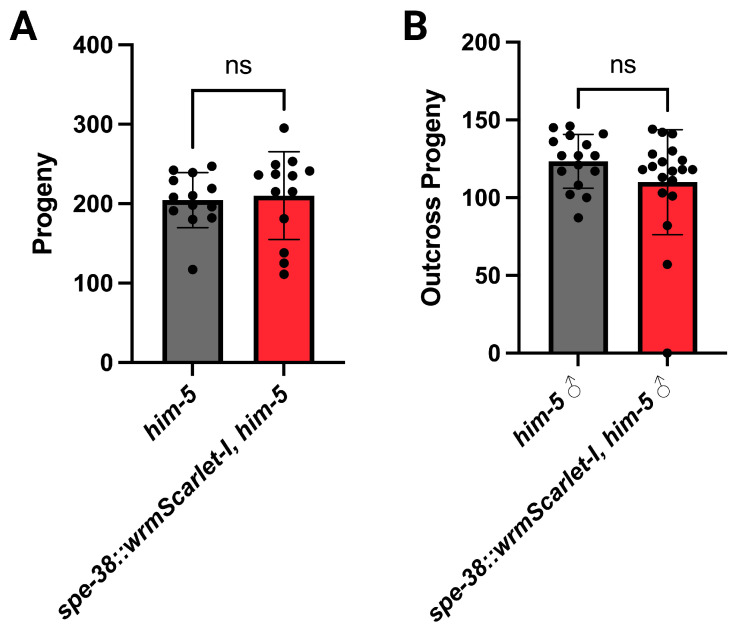
The fertility of genome edited hermaphrodites (**A**) and males (**B**). The fertility of hermaphrodites and males expressing SPE-38::wrmScarlet-I are not significantly different from non-genome edited controls. In all experiments n > 10 animals. The gray (wild-type) and red (genome edited) histogram bars represent the average number of progeny produced. Black dots represent the brood counts for individual hermaphrodites (**A**) or outcross progeny produced (**B**). Error bars represent the standard deviation (SD). For hermaphrodite and male fertility, two-tailed unpaired Student’s *t*-test, *p* > 0.05 indicates not significant (ns).

The wrmScarlet-I fluorescent tag was fused to the C-terminus of endogenous SPE-38 (Figure 1B). When modifying an endogenous gene with a fluorescent tag, there is a risk that the additional amino acids will interfere with normal biochemical activity or interactions of the native molecule. We have previously observed that CRISPR/Cas9 fusions can knock out the activity of other *spe-9* class genes [34]. Backcrossed SPE-38::wrmScarlet-I animals have no major loss of fertility. However, we quantified both hermaphrodite (Figure 2A) and male fertility (Figure 2B). We detect no significant loss of fertility in either hermaphrodites or males. We conclude that the wrmScarlet-I does not interfere with function and these results support the idea that any detected signal likely represents the natural distribution of SPE-38 in spermatids and spermatozoa.

### 3.2. SPE-38::wrmScarlet-I Is Localized to the Membranous Organelles in Spermatids

**Figure 3:**
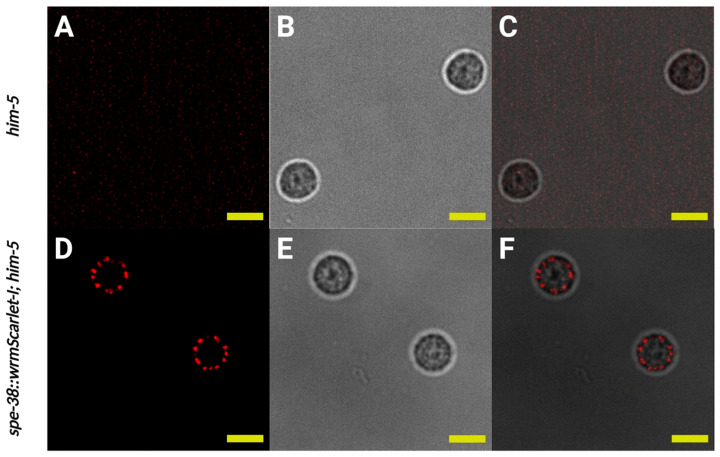
Localization of SPE-38::wrmScarlet-I visualized by red fluorescent signal to membranous organelles (MOs) in spermatids. (**A**–**C**) Imaging of the same control spermatids dissected from *him-5(e1490)* males. (**A**) Single confocal section of control spermatids. (**B**) Bright field image of control spermatids. (**C**) Merge of (**A**,**B**). (**D**–**F**) Imaging of *spe-38::wrmScarlet-I*; *him-5(e1490)* spermatids dissected from males. (**D**) Single confocal section of *spe-38::wrmScarlet-I*; *him-5(e1490)* spermatids. (**E**) Bright field image of *spe-38::wrmScarlet-I*; *him-5(e1490)* spermatids. (**F**) Merge of (**D**,**E**). Yellow scale bars, 5 µm.

Confocal microscopy was used to visualize the localization of SPE-38::wrmScarlet-I in spermatids. Control spermatids did not have any detectable fluorescence (Figure 3A–C). Consistent with antibody localization data from the work of Chatterjee et al., 2005 [5], SPE-38::wrmScarlet-I was localized in the unfused MOs of spermatids (Figure 3D–F). One difficulty in detecting fluorescent tags in *C. elegans* sperm is a high level of autofluorescence (Years of Singson lab unpublished observations!). We optimized our illumination and imaging conditions to minimize interference from autofluorescence. Further, under these conditions, we could not detect any significant signal from wild-type sperm compared to our genome edited SPE-38::wrmScarlet-I (Figure 3A–C).

### 3.3. SPE-38::wrmScarlet-I Redistributes to the Plasma Membrane of the Cell Body and the Pseudopod in Mature Sperm

**Figure 4:**
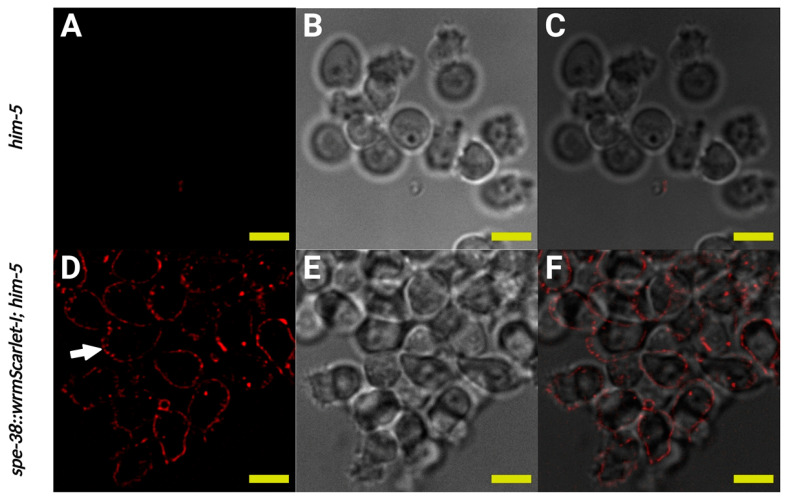
SPE-38::wrmScarlet-I visualized by red fluorescent signal localizes to fused MOs, the cell body and pseudopod plasma membranes in spermatozoa. (**A**–**C**) Imaging of the same in vivo activated control spermatozoa dissected from mated hermaphrodites. (**A**) Single confocal section of control spermatozoa. (**B**) Bright field image of control spermatozoa. (**C**) Merge of (**A**,**B**). (**D**–**F**) Imaging of *spe-38::wrmScarlet-I*; *him-5(e1490)* in vivo activated spermatozoa dissected from mated hermaphrodites. (**D**) Single confocal section of *spe-38::wrmScarlet-I*; *him-5(e1490)* spermatozoa. White arrow indicates fused MOs in a spermatozoa. (**E**) Bright field image of *spe-38::wrmScarlet-I*; *him-5(e1490)* spermatozoa. (**F**) Merge of (**D**,**E**). Yellow scale bars, 5 µm.

SPE-38::wrmScarlet-I localization was determined for in vivo activated spermatozoa. We observed SPE-38::wrmScarlet-I in fused MOs and in the entire plasma membrane including both the cell body and the pseudopod (Figure 4). This localization in live and motile spermatozoa is in stark contrast to the two more limited distributions seen in immunolocalization in differentially fixed spermatozoa (Figure 1C) or see Chatterjee et al., 2005, Figure 8 [5]. When we observe live SPE-38::wrmScarlet-I spermatozoa, we can often see their actively moving pseudopods. We have also observed sperm crawling on the microscope slides. This makes it difficult to capture the cells and pseudopods in exactly the same position when collecting different images over time. We see these same behaviors in similarly treated wild-type sperm not expressing SPE-38::wrmScarlet-I. We can conclude that our isolated SPE-38::wrmScarlet-I spermatozoa have no in vitro behavioral defects compared to wild-type cells. Of significant note, this new localization data suggests that immunolocalization of SPE-38 in mature sperm cannot detect the entire population of the molecule or that fixation protocols lead to artifactual distributions. We favor the unfixed live cell localization data over fixed and nonmoving cells localization data [35].

As we observe the localization of SPE-38 as well as other sperm surface molecules (Singson Lab unpublished observations, [34,36]), we often note slightly more intense signal from the area around the fused MOs compared to the pseudopod membrane (Figure 4D, indicated by white arrow). Transmission electron microscopy ultrastructural studies on wild-type sperm of several nematode species, including *C. elegans* sperm, reveal the highly convoluted structure of unfused and fused MOs [37]. Therefore, there is a high concentration of membrane structure in MOs. If SPE-38 is uniformly distributed in all membranes of fused MOs, the cell body, and the pseudopod, higher amounts of signal could be expected from higher membrane concentrations around MOs in the cell body. Alternatively, differential signal intensity from different sperm structures could represent meaningful areas of differential molecular concentration and a corresponding amount of inherent biological activity associated with SPE-38. For instance, if a molecule was required for sperm-egg binding, one might expect higher concentration where that binding function would be required by the cell. However, a distribution of SPE-38 to the entire plasma membrane of mature spermatozoa puts it in a position to directly interact in trans with egg surface molecules during fertilization.

## 4. Discussion

Loss of *spe-38* gene function has the same sperm sterile mutant phenotype as all other known *spe-9* class of *C. elegans* genes [2,6]. Mutations in the *spe-38* gene lead to failure of sperm to fertilize eggs even though they have wild-type morphology, motility, and behavior. The SPE-38 protein has been found to have the most well-characterized molecular interactions of any of the cloned *spe-9* class of molecules [7,9]. Therefore, it is critical to have the best possible understanding of SPE-38 subcellular distribution in sperm. A diagrammatic summary of the redistribution of SPE-38 from unfused MOs in spermatids to the plasma membrane in live motile spermatozoa is in Figure 1C.

Immunolocalization of SPE-38 gave us two distinct distribution patterns in mature sperm depending on fixation conditions or live cell antibody binding conditions [5]. As reported here, we sought to resolve this localization paradox. Rather than one pattern being incorrect, we conclude that the two patterns rather were incomplete in two different fixation dependent ways. Experimental conditions can lead to artifactual or incomplete results with regards to protein localization and molecular interactions in cells [38]. Taking advantage of new technologies that were not available to us in 2005, we now have a more comprehensive understanding of the subcellular distribution of SPE-38. Our results suggest that our previous localization patterns were incomplete or artifactually concentrated to different cellular regions under different immunolocalization and fixation conditions. A well know example of differential distribution of the same molecule is the detection of G-actin versus F-actin [39,40]. Using a GFP fusion of an actin binding protein, we could detect F-actin in the *C. elegans* fertilization cone, but not any G-actin [41]. In this case, the GFP fluorescent signal only represented a fraction of all actin in the newly fertilized worm embryo.

Why might the anti-SPE-38 antibodies not detect the full localization pattern in mature spermatozoa? Different fixation conditions could possibly alter antibody access to binding epitopes [35]. The local cellular environment on the cell body verses the pseudopod could also differentially alter epitope binding. It has been suggested that the cell body membrane is much less fluid than the pseudopod plasma membrane [42] based on cell surface bound lectin movements. Alternatively, SPE-38 could be in a complex with different binding partners in different regions of the plasma membrane. These binding partners would differentially block antibody epitope access in a fixation dependent manner on the cell body versus the pseudopod. Alternatively, different fixation conditions could cause SPE-38 to artifactually concentrate in either the MOs and the cell body or just the pseudopod plasma membrane. Fixatives such as paraformaldehyde can differentially immobilize molecules depending on cellular context and not provide a representative instantaneous snapshot of distribution in a cell [35].

We believe the localization of SPE-38 on the pseudopod plasma membrane is significant with regards to nematode fertilization. W. Eugene Foor was able to capture amazing images of zygote formation in the nematode *Ascaris lmbricoides* with transmission electron microscopy [43]. In this tour de force work, the pseudopod plasma membrane is clearly the region of the sperm that fuses with the egg plasma membrane. Plasma membrane continuity between the sperm pseudopod and the egg oolemma is clearly visible. The pseudopod would therefore be analogous to the equatorial segment of mammalian sperm [14,44]. If SPE-38 was not localized to the pseudopod membrane of mature sperm, it would be unlikely to play a direct role in sperm-egg interactions.

Previous work demonstrated that SPE-38 is required to allow or promote movement SPE-41/TRP-3 Ca2+ channel protein from MOs to the plasma membrane of mature sperm [7]. It was also shown that SPE-38 and SPE-41/TRP-3 can bind to each other. SPE-41/TRP-3 localizes to unfused MOs in spermatids and localizes to fused MOs, and the plasma membrane of the cell body and pseudopod of mature sperm [11]. If SPE-38 was only localized to the MOs and the cell body plasma membrane, then SPE-38 and SPE-41/TRP-3 would separate at the pseudopod plasma membrane. Our new data now indicates that these two proteins have essentially identical overall localization patterns. Along with binding data [7,9], we believe that that these two molecules have important cis interactions required for a functional fertilization synapse in *C. elegans* and that SPE-38 could have multiple cellular and intercellular functions.

Live cells imaging allows the observation of the dynamic redistribution of molecules. The new worm strain reported here will be useful for genetic interaction studies with other known sperm molecules. For instance, the *spe-10* [45] and *spe-21* (Suryanarayanan, Kruachunas, and Singson unpublished) genes encode DHHC-CRD zinc-finger membrane proteins. These enzymes regulate the membrane association, trafficking, and function of target molecules through lipidation. SPE-38 has been shown to bind to SPE-10 [9]. It will be interesting to determine if SPE-38 is modified by SPE-10 and if *spe-10* mutations alter the cellular distribution of SPE-38.

We had previously established that SPE-38 is localized to MOs by colocalization with the well-established MO marker 1CB4 [5,7]. By extension, co-localization of SPE-38 with other molecules in MOs will also demonstrate localization to this sperm structure. For instance, the SPE-38::wrmScarlet-I strain will be useful in future colocalization and interaction survey studies with other *spe-9* class molecules. Additionally, we had constructed a *C. elegans* sperm transmembrane protein interactome utilizing a split-ubiquitin membrane yeast two-hybrid system [9]. SPE-38 was found to interact physically with numerous other known sperm transmembrane molecules. The localization of most of these SPE-38 interacting molecules is not known. We can now hypothesize by extension of these binding observations that these binding partners will at minimum localize to the MOs and/or the plasma membrane of sperm.

We believe that fluorescent tagging of SPE-38 in live cells is a superior method to determine *C. elegans* sperm protein localization. Strains of worms expressing endogenously expressed fluorescently tagged *C. elegans* sperm molecules will help us address interactions among a growing list of fertilization molecules and could aid in the future discovery of novel mechanisms and components of the fertilization synapse.
